# Pakistan Abolishes Kidney Market and Ushers in a New Era of Ethical Transplantation

**Published:** 2010-11-01

**Authors:** S. A. H. Rizvi, S. A. Anwar Naqvi, M. N. Zafar, Z. Hussain, A. Hashmi, F. Akhtar, M. Hussain, E. Ahmed

**Affiliations:** *Sindh Institute of Urology and Transplantation (SIUT), Civil Hospital, Karachi 74200, Pakistan*

**HISTORY OF TRANSPLANTATION IN PAKISTAN**

Renal transplantation in Pakistan started in 1979 from living related donors in public sector hospitals. Initially, the activity was as low as <50 cases per year but gradually rose to >100 per year by the mid 1990s [1]. Shortage of donors for lack of a deceased donor program and rapidly-developing expertise lead to unrelated commercial transplants in the private sector where the poor and impoverished society were exploited to donate kidneys for US$ 1000 to 2000 [2]. By the year 2000, the number of transplants per year exceeded 1000—more than 70% of which were unrelated commercial donors [1,2]. Although initially, the majority of the recipients were local, the changing scenario in the region lead Pakistan to become the largest center of transplant tourism by the year 2005—where almost 1500 foreigners received transplants every year in private sector hospitals [3].

Before Pakistan, India was the center of transplant trade and tourism where activity had increased to over 4000 transplants a year. However, after over a decade of this unethical practice, India prohibited kidney trade and tourism in 1994 [4,5]. The background to tourism in Pakistan was firstly the prohibition in India, secondly the absence of law prohibiting sale of organs and transplant tourism, and thirdly transplant centers access to the world by advertising on the Web [6]. Rich buyers traveled to Pakistan from Europe, the Middle East and India and transplant tourism became an industry as foreigners would pay US$ 20,000 to 30,000 for a transplant package [7,8]. This rampant trade exploited the poor where the middle man siphoned most of the money. By the year 2007, of 2500 transplants performed annually in Pakistan, 2000 were unrelated commercial transplants, 1500 of which were for foreigners. Of the 500 living related transplants, 50% were performed in one center in Karachi, southern Pakistan [1].

Pakistan has an estimated prevalence rate of patients with end-stage renal disease (ESRD) of 100 per million population. Of the ESRD patients, about 10% have access to dialysis mostly in the private sector and the transplant rate is 8–10 cases per million population [1,9]. Maintenance dialysis costs US$ 20–25 per session, but transplantation costs US$ 6000–10,000 which is beyond the reach of a common man, as the *per capita* income in Pakistan was between US$ 400 and 600 in the 90s and almost US$ 1000 by the present day estimates [10,11]. Dialysis and transplantation was available for few select people who could afford it with >90% of population disfranchised as more than 50% lived below the poverty line on less than US$ 1 a day [10]. This situation made transplantation irrelevant to the common man and therefore for it to gain ground as a successful therapy, it had to be made available to this population and thereafter the society could be asked to support transplantation, promulgation of law and organ donation from living and deceased. 

**TRANSPLANTATION FOR THE DISFRANCHISED: A MODEL FOR DEVELOPING COUNTRIES**

In developing countries for changes to take place, besides awareness one needs practical examples which are sustainable for long periods. Our Institute in Karachi established a model for transplantation in 1980s. It started an integrated dialysis and transplant program; all services were offered free of charge to all patients to include recipient and donor follow-up and post-transplant immunosuppressive drugs. This program was based on a philosophy of public-government partnerships with the public contributing up to 60%–70% and the government the rest [1,9]. Dialysis and transplant was offered to all and as the funds increased so did the activity from 10 cases per year in 1987 to 544 per year in 2009. Renal transplantation requires back up of dialysis facilities—more the dialysis, more the transplant. The number of dialysis at Sindh Institute of Urology and Transplantation (SIUT) was increased from 200 patients per day in 2005 to 600 per day in 2009. In living related donation, seeing that a beloved one is in severe distress and on the verge of dying results in altruistic donation from the family members. With any delay in transplantation, the early fears of mortality dissipate away and resistance to donation develops within the family. Since dialysis was available freely, transplantation gained firm roots in the public as it was transparent, free and with excellent results, one-year graft survival of 95% and five-year survival of 85% [1,12].

Equally important to recipient survival is the well-being of the donors who donate for no benefit to themselves but for self-esteem and altruism. A donor follow-up clinic with hundreds of healthy rehabilitated donors became a testimony to the success and acceptance of transplantation as a mode of therapy and stimulated others to donate [13]. Excellent results of the Institute established credibility abroad and lead to the first cadaver kidney transplant in Pakistan by the courtesy of Euro-transplant Foundation [14]. Seminars, debates and awareness program brought forward clergy, judiciary and media to support transplantation. After transplantation of 26 deceased donor kidneys from Euro-transplant came the first local deceased donor in 1998 followed by another in 2005. These deceased donor transplants were performed in the absence of the transplant law as the public has been motivated to donate both living related and deceased donors.

**THE ROAD TO TRANSPLANT LAW IN 2010**

The bill on transplantation was introduced in the parliament almost 15 years ago in the early 90s. It remained dormant for the first decade in various specialist committees from government to government mainly due to non-acceptance of transplantation by the society as a therapeutic modality due to social, cultural and religious issues. One of the main reasons was also the lack of dialysis and transplantation in the public sector. This absence of facilities in the presence of expertise moved transplantation in the private sector largely based on living related transplants. Commerce caused a paradigm shift from related to unrelated transplants and the subsequent establishment of the organ bazaar. SIUT, having established itself as the premier transplant institution in the country, started a campaign against organ sale and transplant tourism. The whole society including media, judiciary, clergy, and medical profession was galvanized and took support from the international bodies like World Health Organization (WHO) and The International Transplantation Society (TTS).

**EXPOSING EXPLOITATION AND PLIGHT OF THE VENDORS**

SIUT in collaboration with WHO and TTS held several seminars and symposia to highlight the benefits of deceased donors, care of the living donors and the exploitation and neglect of the kidney vendors. Taking lead from the Amsterdam forum for the care of the donors [15], a socioeconomic survey of the kidney vendors was undertaken in the North of Pakistan. It showed that 34% of the vendors lived below poverty line on less than US$ 1 a day; 90% were illiterate and 69% were bonded laborers who were virtual slaves to the landlords [6]. This modern day slavery is practiced in a number of countries in South Asia where generation after generation of workers suffer long hours to repay debt of their fathers or grandfathers [16]. The majority of the vendors (93%) sold kidneys to pay off debts however, 88% of them had no economic benefits post-vending. Similar findings on vendors have been reported from India [5]. A second study in the same area was conducted in the middle 2007 to evaluate the health status and renal function of kidney vendors. This study showed that vendors as compared to carefully selected living related donors had poor selection as 27% of them were hepatitis C positive and 7.7% had hepatitis B; hypertension was found in 17% of them; they had a mean GFR of 70 mL/min with a third having GFR <60 mL/min putting them at high risk of developing chronic kidney disease [17]. The exploitation of the vendors often lead to court cases against doctors performing organ trade [18] and this in turn lead to judicial activation with *suo motto* notice from the Supreme Court of Pakistan [19]. These findings on the exploitation of the poor vendors, transplant of foreigners, pressure from WHO, TTS, Transplantation Society of Pakistan, Pakistan Society of Nephrology, Pakistan Association of Urological Surgeons together with journalists and media and other NGOs finally pressurized the government to promulgate “The Transplantation of Human Tissues and Organs Ordinance 2007” in November 2007. 

**SALIENT FEATURES OF THE TRANSPLANT ORDINANCE**

The transplant ordinance banned commercial unrelated transplantation of locals as well as foreigners. It allowed donation from living donors who are “first degree relatives” and legally related. In case no donor is available, a “non-first degree relative” can donate after approval by an evaluation committee. The ordinance also allowed donation from “brain dead” donors who had given consent in life to be donors or with the consent of their head of kin. The ordinance established a Human Organ Transplant Authority whose job was to identify and recognize transplant centers to perform transplants from living and deceased donors. Each center has to have an evaluation committee with two notables of the society along with medical specialists. Each of the four provinces has to have a Provincial Monitoring Authority who would inspect centers within the province regularly and vet their records of transplants. All transplants have to be reported to the National Monitoring Authority and be part of the National Registry. Transplants have to be reported within 48 hours through a preset pro-forma giving details of recipient and donor, their national identity card copies, relationship and signature of the Hospital Monitoring Authority Chairperson. Finally, punishment for contravening the ordinance was 10 years imprisonment and a fine of U$ 15,000.

**POST-TRANSPLANT ORDINANCE SCENARIO IN PAKISTAN**

The transplant ordinance has almost put an end to exploitation of the poor as commercial unrelated transplants of foreigners and local have come to negligible numbers and there have been several arrests of those performing clandestine operations in back alley hospitals [20]. The Monitoring Authority has given recognition to 44 hospitals throughout the country to perform transplantation. Six months after the ordinance the National Monitoring Authority reported 431 renal transplants. Meanwhile, those performing commercial unrelated transplants have started a rumor campaign that transplant activity has gone down and patients are dying of want of donors [21]. 

The facts are contrary since before the ordinance the total number of transplants estimated in the country was about 2500 per year. Of these, 1500 were foreigners, 500 local unrelated and 500 living related transplants. Although 431 transplants have been performed during six months after ordinance, there has been a resurgence of ethical transplants in the country. Several ethical centers have taken up the challenge with SIUT taking the lead. It has increased transplants from three per week before the ordinance to 10 to12 per week, after the ordinance taking the numbers to 544 in 2009. This has made SIUT the only center in the world performing the maximum number of living donor renal transplants in a year [22]. The equation of transplantation in Pakistan has been balanced, since by the end of the year 2008 over 1000 more transplants of locals (compared with that of the pre-ordinance period) have been performed. According to Human Organ Transplant Authority, the number of transplants in 2009 were 871 cases—544 (62%) of which were performed at SIUT.

Soon after passing the legislation, the powerful commercial lobby started a campaign against the law implying that it was restrictive and patients were dying of shortage of organs (actually unrelated commercial donors). A sinister move was made to bring amendments in the ordinance in the National Assembly to permit unrelated donors to be paid by the recipients and to allow transplant of foreigners (to help economy of the country!). A committee of the National Assembly rejected these amendments in January 2009 and the ordinance became a law and part of the Constitution of Pakistan. The failure did not deter the commercial lobby and they moved the Sharia Court.

The Federal Sharia Court is an affiliate bench of the Supreme Court of Pakistan which ensures that all laws in Pakistan are in accordance with the Islamic laws. Recently, the Society of Transplant Physicians and Surgeons of Pakistan, representing those who were involved in commercial transplants, moved a petition in the Sharia Court that certain provisions of the “Transplantation of Human Organs and Tissue Ordinance 2007” were against Islamic laws. The petitioners pleaded that the law was restrictive and discriminatory as only close blood relatives were allowed to donate. Furthermore, they pleaded that the law is discriminatory as it forbids transplant of Muslims from other countries.

The Federal Government supporting the legislation was represented by the Administrator of Human Organ Transplant Authority. The government was assisted by the Transplantation Society of Pakistan, Pakistan Nephrology Society and Pakistan Association of Urological Surgeons as well as members of Human Right groups. The President of the Transplantation Society of Pakistan appeared as *amicus curiae* for the societies to oppose the petitioners. The Sharia Court, after holding eight hearings in three cities of Pakistan, dismissed the petition. The Court declared that “sale or purchase of human organs and transplant of foreigners who did not have legitimate family donors is against the spirit of Islamic laws.”

**THE WAY FORWARD**

In March 2010, the President of Pakistan signed the bill for organ transplantation and it became the part of the constitution of Pakistan. The bill made history as it was passed unanimously by the two houses of legislature—the National Assembly and the Senate.

In Pakistan, transplantation is now accepted as a successful therapeutic modality for end-stage organ failure. The last 20 years have shown that society can be motivated to donate and sustain transplantation when it is available to all who need it and cannot afford to pay. Legislation is the key to check commercialism in developing countries. It may be weakly implemented but is a strong deterrent as shown in our country. Dialysis and transplant should be fully integrated and carried out in the public sector hospitals. Finances could be provided on a model of public-government partnership while the doctors act as catalyst [1,9]. There is need for comprehensive follow-up of both recipient and donors with the provision of immunosuppressive drugs. New dialysis and transplant centers should be established in the interior of the country so that the society will be able to avail transplantation. Our experience has shown that making transplant available to the non-affording people not only increases acceptance but also motivates the society to donate. We have thus been able to increase our activity from three cases per week in pre-ordinance period to 10–12 cases per week post-ordinance and have taken the burden of other centres so that currently, over 40% of transplants are come from other provinces. This increased availability of transplantation is more likely to open the door to a viable deceased donor program in Pakistan. The first post-law local deceased donor transplants were also performed at SIUT in April 2008. In March 2010, the second deceased donation took place in Islamabad, Pakistan. One kidney was transplanted in Islamabad and the second was flown to Karachi at SIUT, 1000 miles away for transplantation—the beginning of organ sharing in Pakistan.
